# Demographic shifts reshaping the landscape of hand trauma: a comprehensive single-center analysis of changing trends in hand injuries from 2007 to 2022

**DOI:** 10.1186/s40621-024-00510-8

**Published:** 2024-06-13

**Authors:** Martynas Tamulevicius, Florian Bucher, Nadjib Dastagir, Vincent Maerz, Peter M. Vogt, Khaled Dastagir

**Affiliations:** Department of Plastic, Aesthetic, Hand and Reconstructive Surgery, Hannover Medical School, Carl-Neuberg-Str. 1, 30625 Hannover, Germany

**Keywords:** Hand injuries, Demographic changes, Aging, Hand emergencies, Hand trauma

## Abstract

**Introduction:**

Hand injuries constitute up to 30% of the total cases treated in emergency departments. Over time, demographic changes, especially an aging population, and shifts in workplace safety regulations and healthcare policies have significantly impacted the landscape of hand trauma. This study aims to identify and analyze these evolving trends over nearly two decades.

**Methods:**

In this retrospective, cross-sectional study, we investigated patients who were admitted to the high-volume regional hand trauma center of a university hospital between January 2007 and December 2022. We analyzed trends in patients’ demographics and annual alterations of injuries. For the comparative analysis, patients were divided into two groups based on the time of presentation: the early cohort (2007–2014) and the current cohort (2015–2022).

**Results:**

A total of 14,414 patients were admitted to our emergency department within the study period. A significant annual increase in patient age was identified (R^2^ = 0.254, *p* = 0.047). The number of presentations increased annually by an average of 2% (*p* < 0.001). The incidence of the following hand injuries significantly increased: sprains/strains (+ 70.51%, *p* = 0.004), superficial lacerations (+ 53.99%, *p* < 0.001), joint dislocations (+ 51.28%, *p* < 0.001), fractures (carpal: + 49.25%, *p* = 0.003; noncarpal: + 39.18%, *p* < 0.001), deep lacerations (+ 37.16%, *p* < 0.001) and burns and corrosions (+ 29.45%, *p* < 0.001). However, rates of amputations decreased significantly (− 22.09%, *p* = 0.04).

**Conclusions:**

A consistent and significant annual increase in both the total number of injuries and the average age of patients was identified. An aging population may increase injury rates and comorbidities, stressing healthcare resources. Our study underscores the need to adapt healthcare structures and reimbursement policies, especially for outpatient hand injury care.

**Supplementary Information:**

The online version contains supplementary material available at 10.1186/s40621-024-00510-8.

## Background

Hand injuries are common and account for a significant number of emergency department visits and hospital admissions. The prevalence of hand and wrist injuries lies between 10 and 30% of all presentations in the emergency department (Robinson and O'Brien 2019; Angermann and Lohmann 1993; Larsen et al. 2004; Rosberg and Dahlin 2004; Hill et al. 1998; Dubert et al. 2010; Leerdam et al. 2022). The impact of these injuries on individuals and healthcare systems is significant, as they can result in significant pain, disability, and loss of productivity. Moreover, more severe injuries are frequently associated with delayed recovery and the potential risk of long-term disability, followed by significant costs to the patient and healthcare system (Robinson and O'Brien 2019; Rosberg et al. 2013; Şahin et al. 2013; Trybus et al. 2006).

In recent years, there have been significant changes in the demographics and number of patients presenting with hand injuries to emergency departments. Current literature reports conflicting and very limited findings regarding alterations in the number of hand injuries over the past decades. Recent studies from Europe tend to report increasing numbers, while studies from America have reported significant decreases (Stang et al. 2021; Manley et al. 2019; Gordon et al. 2021; Shah et al. 2012). Unfortunately, these studies focused on a selected number of injuries, restricting our ability to grasp the comprehensive scope of hand injuries in trauma centers. Furthermore, there is a considerable lack of information on their association with demographic shifts in the population and their potential impact on the division of healthcare resources. According to the World Health Organization, in 2019, there were 1 billion people aged 60 years and older. By 2050, this number is projected to reach 2.1 billion (Stang et al. 2021). This rapid increase, especially in developing nations, underscores the demographic shift toward an aging population.

The purpose of this study was to evaluate the trends and treatment strategies for a broad spectrum of hand injuries in a high-volume hand trauma center of a university hospital over 16 years. Through this analysis, we aim to understand the impact of demographic changes on these injuries and identify potential areas for improvement in the management of such injuries and the allocation of healthcare resources.

## Materials and methods

### Study design

In this retrospective, cross-sectional descriptive epidemiological study, we investigated patients who, between January 2007 and December 2022, presented to the emergency department of Hannover Medical School (Hannover, Germany), a high-volume university hospital, with one of the following acute hand injuries:

1. Superficial lacerations (shallow cuts or wounds that only affect the outermost layers of the skin or that do not penetrate deeper tissues or structures (subcutaneous tissue, muscles, tendons, nerves, etc.), including contusion, abrasion, insect bite, superficial foreign body);
2. Deep lacerations (including one of the following: injury to nerves, blood vessels (arteries or/and deep veins), the articular capsule, muscles or tendons);
3. Complex hand injuries requiring complex surgical intervention (including open wounds with a combination of at least two of the following injuries: fractures, injury to nerves, blood vessels, articular capsule, muscles or tendons);
4. Amputations;
5. Wrist fractures;
6. Metacarpal and finger fractures;
7. Joint dislocations;
8. Acute joint inflammations;
9. Sprains and strains;
10. Phlegmon of the hand (including Paronychia and Panaritium);
11. Hand and wrist tenosynovitis;
12. Hand burns and corrosions.

Patients who presented with the same injury were excluded from the analysis.

A comparative analysis of patient demographics, incidence and type of injury, treatment strategy and clinical course between injuries annually was performed. Using descriptive analyses, trends in the number of injuries over time were identified. For additional comparative analysis, we divided the patient cohort into two groups: the early cohort (EC) for the period between January 2007 and December 2014 (8 years) and the current cohort (CC) for the period between January 2015 and December 2022 (8 years). Patient demographics, clinical course, and the incidence and nature of injuries were compared between the groups.

### Statistical analysis

Values are presented as the mean ± standard deviation, except in graphs depicting age trends, where the mean ± standard error of the mean is utilized. Categorical variables are reported as numbers and percentages. Statistical analysis was performed using SPSS version 27.0 (IBM^®^ SPSS^®^-Software platform for Windows) and GraphPad Prism version 9.5.1 (GraphPad Software for Windows, San Diego, California, USA). A value of *P* < 0.05 was considered significant. Pearson’s correlation was used to determine correlations between continuous variables. Dichotomous variables were compared using Pearson's chi-squared test. Linear regression analysis was used to analyze the significance of changes in the trends of injuries over time.

### Data source

All the data were assembled and analyzed retrospectively and anonymously. The initial data gathering took place within the institutional Enterprise Clinical Research Data Warehouse (ECRDW) after approval was obtained from the local healthcare data protection commission. Ethical approval for this study was waived by the local ethics committee.

## Results

### General trends of injuries

Over the study period, a total of 14,414 patients who experienced hand injuries presented to our emergency department. A total of 14.0% of patients had more than one hand injury at presentation to the emergency department. In the past eight years, the rate of admission to the emergency department has increased by 56.13% (from 5628 to 8787 patients). The number of injuries has been steadily increasing at an average rate of 2% per year, which translates to an additional 47 cases annually (R^2^ = 0.792, *p* < 0.001).

### Age and gender trends

The mean age of all patients was 40.09 ± 19.58 years (range, < 1 to 100 years). Most of the patients were males in both the adult and nonadult groups (n = 9274, 64% and n = 1055; 65.5%, *p* ≤ 0.001 for both groups). Females were significantly older than males (40.93 ± 20.28 vs. 39.63 ± 19.17, *p* ≤ 0.001). There were 1610 (11.17%) patients who were nonadults or younger than 18 years in our study cohort, with a mean age of 10.25 ± 5.29 years. However, in the nonadult group, females were significantly younger than males (9.61 ± 5.47 vs. 10.60 ± 5.16, *p* < 0.001) (Table 1).

**Table 1:** Patient demographics

Category	All patients	Females	Males	*P* value	All nonadult* patients	Females of all nonadult patients	Males of all nonadult patients	*P* value**
Gender (n)	14.414	5140 (36%)	9274 (64%)	< 0.001	1610 (11.17%)	555 (35.5%)	1055 (65.5%)	0.152
Age (years)	40.09 ± 19.58	40.93 ± 20.28	39.63 ± 19.17	< 0.001	10.25 ± 5.29	9.61 ± 5.47	10.60 ± 5.16	< 0.001

^*^Nonadult patients were defined as those < 18 years of age

^**^Between adult and nonadult patients

**Fig. 1:**
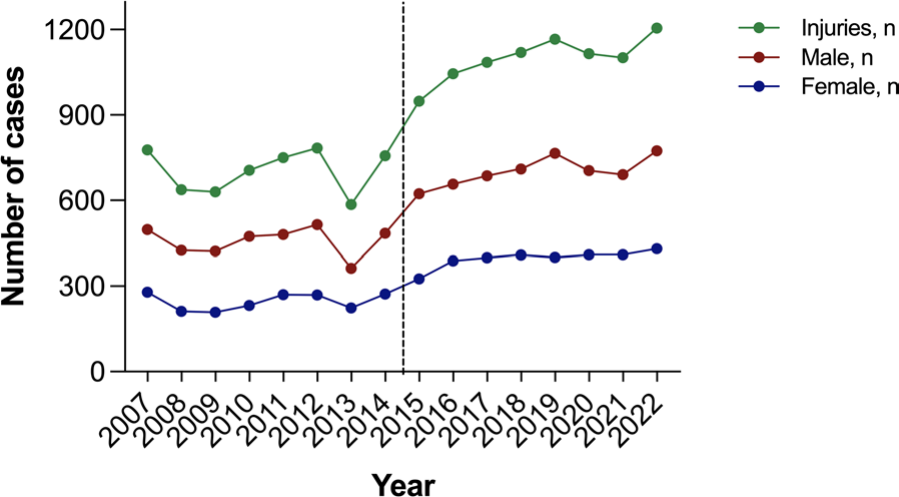
Trends in injuries and gender-based distribution

**Fig. 2:**
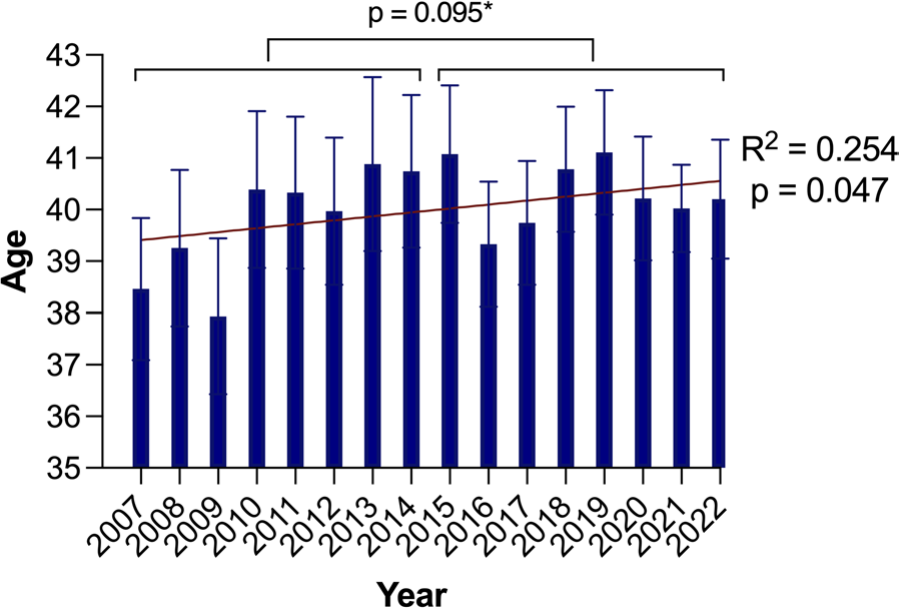
Age trends in all patients. *p value demonstrates the age difference between the early cohort and the current cohort

**Fig. 3:**
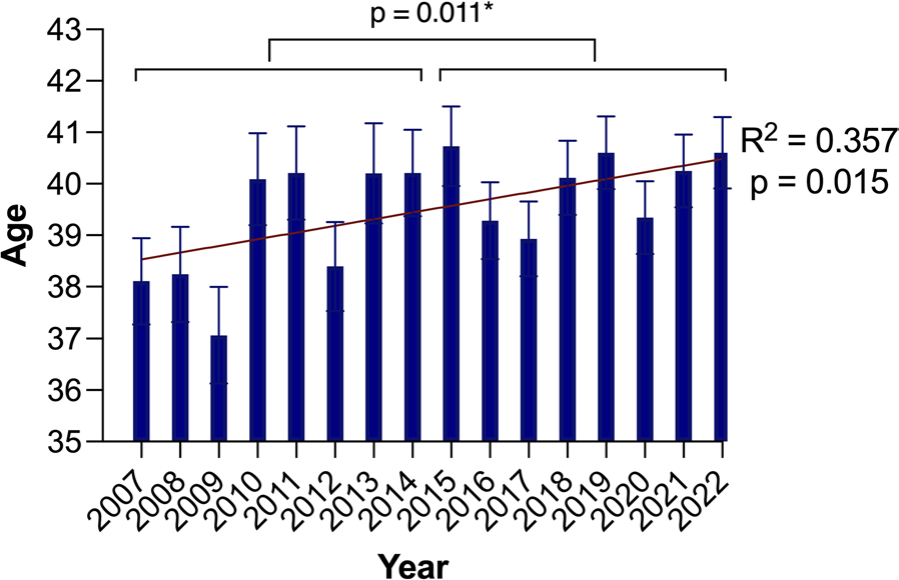
Age trends in men. *p value demonstrates the age difference between the early cohort and the current cohort

**Fig. 4:**
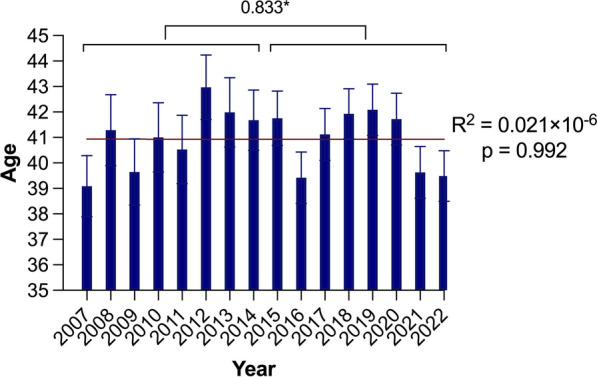
Age trends in women. *p value demonstrates the age difference between the early cohort and the current cohort

Throughout the analyzed years, the majority of patients remained male, and there was no significant difference in the male-to-female ratio between the groups (1.86 vs. 1.77, *p* = 0.15) (Fig. 1). The average age of patients in the CC was slightly higher, and a weak but significant annual trend was identified (R^2^ = 0.254, p = 0.047), even though the difference was not statistically significant (39.75 ± 19.61 vs. 40.31 ± 19.56, *p* = 0.095) (Fig. 2). However, the mean age of the males in the CC was significantly greater, and a weak but significant annual trend could be identified (39.06 ± 19.15 vs. 39.99 ± 19.18, *p* = 0.011; R^2^ = 0.357, *p* = 0.015) (Fig. 3 and 4).

A statistically significant increase in hand injuries between the early cohort and current cohort was observed in all age groups, except for the age group between 0 and 9 years (*p* = 0.77). The highest increases were observed in the following age groups: 90+ years (+ 78.85%), 50–59 years (+ 43.27%), 80–89 years (+ 43.10%), 20–29 years (+ 41.38%) and 30–29 years (+ 41.30%). Regression analysis demonstrated a significant annual increase in nearly all age groups, except for those aged 0–9 years (Table 2). The age groups with the highest coefficients of determination and therefore significant trends were as follows: 50–59 years (R^2^ = 0.83, p < 0.001), 60–69 years (R^2^ = 0.78, p < 0.001), 20–29 years (R^2^ = 0.73, *p* < 0.001) and 30–39 years (R^2^ = 0.71, *p* < 0.001) (Table 2).

**Table 2:** Annual rates of all hand and wrist injuries by age cohort

	2007	2008	2009	2010	2011	2012	2013	2014	2015	2016	2017	2018	2019	2020	2021	2022	Total	Change between EC and CC (%)*	*P* value	R^2^	*P* value**
0–9	41	37	46	41	45	49	28	37	35	50	51	37	48	40	33	38	656	+ 2.41	0.77	0.01	0.75
10–19	87	76	52	63	87	81	46	54	76	90	118	93	121	119	132	143	1438	+ 38.79	**< 0.001**	0.58	**< 0.001**
20–29	149	101	135	127	138	140	113	144	181	245	224	238	213	224	222	239	2833	+ 41.38	**< 0.001**	0.73	**< 0.001**
30–39	148	99	107	118	106	124	90	139	190	189	177	218	198	200	199	215	2517	+ 41.30	**< 0.001**	0.71	**< 0.001**
40–49	138	134	120	123	134	135	116	131	169	134	161	181	173	165	163	151	2328	+ 20.51	**< 0.001**	0.50	**0.002**
50–59	93	91	84	108	96	115	81	120	131	172	177	160	192	178	166	213	2177	+ 43.27	**< 0.001**	0.83	**< 0.001**
60–69	72	57	47	63	65	70	65	77	81	89	90	84	119	98	104	105	1286	+ 32.99	**< 0.001**	0.78	**< 0.001**
70–79	30	30	29	41	49	44	35	40	50	52	54	65	68	56	41	58	742	+ 32.88	**< 0.001**	0.58	**< 0.001**
80–89	18	10	10	21	29	22	11	15	31	16	30	37	32	28	33	32	375	+ 43.10	**0.001**	0.51	**0.002**
90 +	1	2	1	1	1	4	1	0	4	8	3	8	3	7	8	11	63	+ 78.85	**< 0.001**	0.62	**< 0.001**
Total	777	637	631	706	750	784	586	757	948	1045	1085	1121	1167	1115	1101	1205	14,415	+ 56.13	**< 0.001**	0.79	**< 0.001**

Bold: A value of *P* < 0.05 was considered significant

^*^Change in frequency between the early cohort and the current cohort

^**^P value for the significance of linear regression analysis

### Trends of different types of injuries

**Fig. 5:**
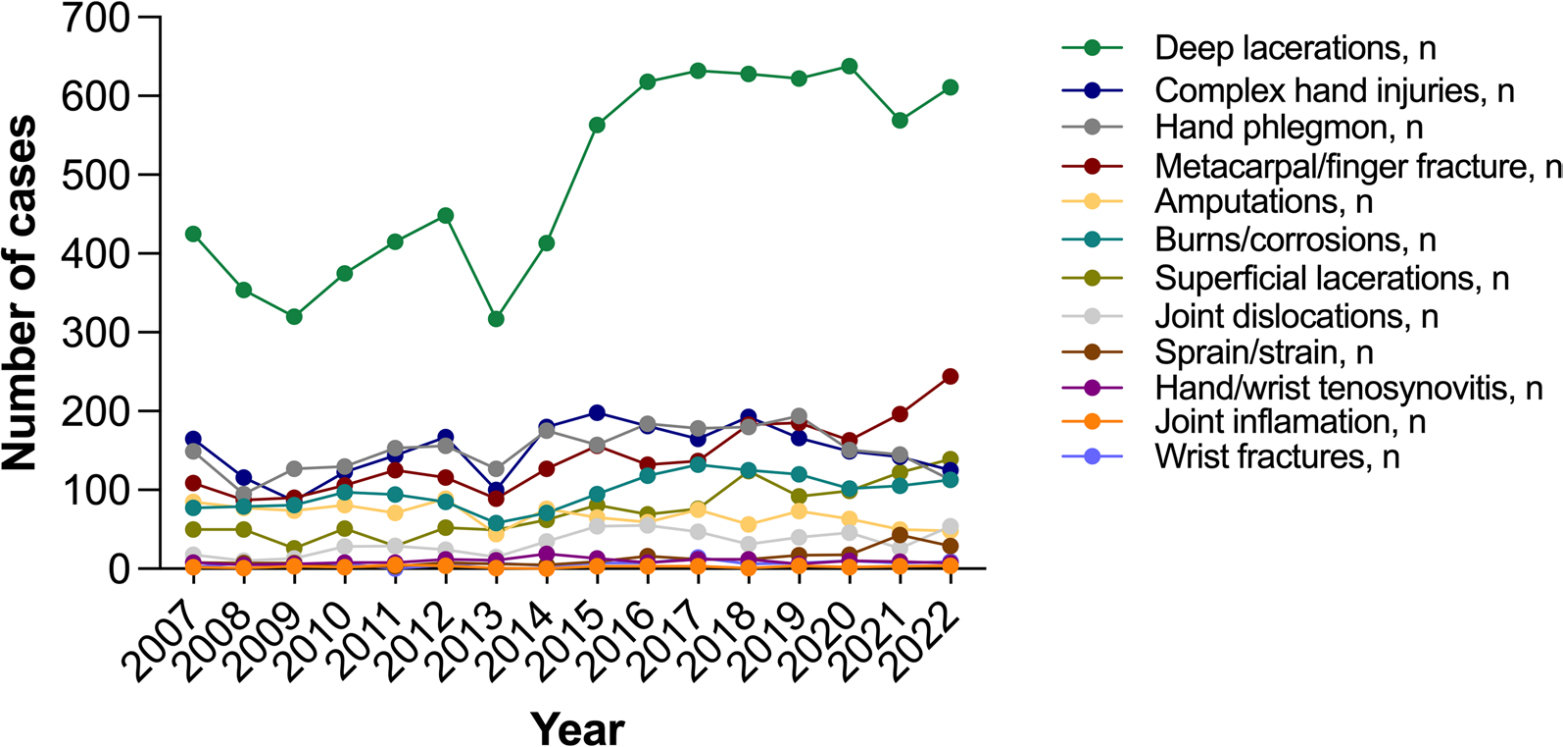
Annual changes in different types of injuries

The most common injuries were deep lacerations (n = 7948, 40.84%), hand phlegmon (n = 2414, 12.17%), complex hand injuries (n = 2400, 12.10%), burns and corrosions (n = 1552, 7.82%), superficial lacerations (n = 1171, 5.90%) and amputations (n = 1086, 5.47%) (Fig. 5). According to the comparative analysis between the EC and CC, the following hand injuries significantly increased in the CC: sprains and strains (+ 70.51%, *p* = 0.004; R^2^ = 0.586, *p* < 0.001), superficial lacerations (+ 53.99%, *p* < 0.001; R^2^ = 0.788, *p* < 0.001), joint dislocations (+ 51.28%, *p* < 0.001; R^2^ = 0.473, *p* < 0.001), wrist fractures (+ 49.25%, *p* = 0.003; R^2^ = 0.364, *p* = 0.013), metacarpal and finger fractures (+ 39.18%, *p* < 0.001; R^2^ = 0.784, *p* < 0.001), deep lacerations (+ 37.16%, *p* < 0.001; *R*^2^ = 0.702, *p* = 0.003) and burns and corrosions (+ 29.45%, *p* < 0.001; *R*^2^ = 0.429, *p* = 0.006). According to the same analysis, the rate of amputation decreased significantly (− 22.09%, *p* = 0.04; R^2^ = 0.415, *p* = 0.007). However, complex hand injuries, phlegmon of the hand, hand and wrist tenosynovitis, and acute joint inflammation remained unchanged (all *p* ≥ 0.068) (Supplementary Table 1). A statistically significant decrease in the rate of amputations was observed within the following age groups: 0–9 years and 30–39 years (Supplementary Table 2). Conversely, there was a statistically significant increase in the incidence of burns and corrosions among adults aged 20 to 69 years (Supplementary Table 3). In all age groups beginning at 10 years and older, a significant increase in the incidence of deep lacerations and metacarpal and finger fractures was noted, particularly among individuals aged 20 to 39 and those aged 70 and above for deep lacerations and among those aged 10 to 29 and 70 to 89 for the aforementioned fractures (Supplementary Tables 3, 4). A similar significant increase was observed in the age group of 10–29 years for sprains and strains, in the age group of 10–59 years for superficial lacerations, and in the age group of 10–69 years for wrist fractures (Supplementary Tables 5–7).

## Discussion

In our analysis of hand injuries spanning nearly two decades, we have observed a significant demographic shift towards an aging population (R^2^ = 0.254, *p* = 0.047). This shift was predominantly observed in the male population (R^2^ = 0.357, *p* = 0.015), which comprised two-thirds of all patients admitted to our hand trauma center. Additionally, we observed a notable increase in emergency department visits among the elderly patient group aged over 80 years. This could be attributed to the growing elderly population. According to a demographic analysis of the region primarily served by our hand trauma center, the population has shown consistent growth over the last decade. Between the end of 2008 and the end of 2018, it grew by 60,500 people (+ 5.4%), reaching 1.18 million from 1.12 million Based on the same analysis, the population of elderly individuals aged 65 and above has risen by 6.0 percent, comprising nearly one-fourth of the region's total population (Landeshauptstadt Hannover und Region Hannover 2020). This data corresponds to the national trends reported by the National Statistical Office of Germany, which indicates that the number of people aged 67 and older increased by 54%, from 10.4 to 15.9 million, between 1990 and 2018 (Statistisches Bundesamt 2019). The World Health Organization also reports similar significant increases in the proportion of people aged 60 years and older in the global population (Stang et al. 2021). Elderly individuals, who often have multiple comorbidities, are at greater risk for hand trauma. Studies have shown that approximately one-fourth of trauma patients who visit an emergency department are over 65 years of age, and those with high comorbidity are more likely to experience higher mortality rates (Manley et al. 2019; Gordon et al. 2021). Furthermore, Rosberg et al. reported an increase in the number of hand injuries in the elderly population over 30 years (World Health Organization 2024). Some authors have reported controversial data. Their findings indicate that while the incidence of hand injuries among elderly individuals may be lower compared to younger demographics, elderly men exhibit a higher susceptibility to injuries resulting from hazardous equipment, whereas elderly women are at a greater risk of sustaining fractures following a fall (Statistisches Bundesamt 2019; Keller et al. 2012; Perdue et al. 1998). In our analysis, we have observed substantiating data indicating that among the growing elderly population, the most common injuries with the highest increases in the current cohort were amputations, deep lacerations, and fractures, even though we did not specifically analyze the etiology of specific injuries (Supplementary Tables 2–7). Additionally, these hand injuries in elderly patients tend to be more severe and require more complex treatment (Rosberg and Dahlin 2018).

We also observed statistically significant increases in emergency department visits between the EC and CC in almost all age groups, except for the group of pediatric patients aged between 0 and 9 years (*p* = 0.77). The highest increases were observed annually among working-age adult males (aged between 20 and 59 years), who also experienced hand and wrist injuries nearly twice as frequently as females, despite females comprising the majority of the population in the region (50.8%) (Landeshauptstadt Hannover und Region Hannover 2020). Similar findings were observed in the analysis of patient cohorts in other populations (Keller et al. 2012; Kringstad et al. 2019; Sayyari et al. 2022). We noted a significant shift at the age of 70, with females constituting two-thirds of the total patients aged 90 and above. This phenomenon can be attributed to the notably lower life expectancy among men compared to women, with figures of 78.5 years versus 83.0 years in our region and 78.2 years versus 83.2 years in Germany (Trybus et al. 2004; Landeshauptstadt Hannover und Region Hannover 2020). The World Health Organization corroborates this trend, reporting comparable data for the European region: a life expectancy of 75 years for men and 81 years for women (World Health Organization 2024).

In our analysis, we observed a steady rise in admissions, averaging an annual increase of approximately 2%. Over the past 8 years, there has been a striking 56.13% surge in admissions compared to the preceding 8 years. However, the current literature presents conflicting findings regarding the global trend in the number of hand injuries. Gordon et al. recently conducted an analysis of a nationwide database in the United States evaluating wrist, hand, and finger injuries. They reported an 8.6% decrease in all visits to the emergency department from 2009 to 2018 (Kringstad et al. 2019). Conversely, a 17 years analysis in England (between April 1998 and March 2015) demonstrated a 57% increase in the incidence of hand injuries (Keller et al. 2012). Similar findings were reported in a single-center study conducted by Stang et al., indicating a substantial rise in emergency department visits from around 300 patients in 2009 to approximately 1100 patients in 2018 (Plotsker et al. 2022). The majority of injuries contributing to the increase in our patient cohort could be attributed to a significant increase in emergency department visits for specific injury types, including superficial lacerations, deep lacerations, fractures, sprains, strains, and joint dislocations. These findings align with those reported in the aforementioned studies (Keller et al. 2012; Plotsker et al. 2022). The majority of these injuries were minor in nature and could be managed in smaller clinics or even private medical centers. Although some patients may require some form of surgical treatment. A consensus paper released by the German Society for Hand Surgery in 2020 affirmed that under specific circumstances, approximately 80% of procedures can be effectively conducted on an outpatient basis (Trybus et al. 2004). This pattern of increasing numbers of visits to the emergency department with minor hand and wrist injuries is possibly a result of the constantly decreasing number of clinics that treat acute hand injuries in our region. Unfortunately, the healthcare system's current structure in Germany and compensation for outpatient procedures have not been adapted, making them less appealing to smaller profit-based hospitals or private medical clinics (Jasilionis et al. 2023). Another contributing factor to the potential increase in patient numbers at hand trauma centers in the future is the aging workforce among out-of-hospital practicing physicians. According to the German Medical Association, 41% of these physicians are over 60 years old, with nine percent aged 65 years or older, often not fully available to the job market (Bundesärztekammer 2023).

The most common injuries in our patient cohort were superficial lacerations, deep lacerations, complex hand injuries, amputations, metacarpal and finger fractures, phlegmon of the hand and burns and corrosions of the hand. To date, no published studies have analyzed this broad spectrum of hand injuries in patients who presented to the emergency department of a high-volume hand trauma center. Gordon et al. reported similar rates of lacerations (48.6% vs. 46.74%), higher rates of fractures (19.9% vs. 11.83%) and strains and sprains (1.02% vs. 12.3%) (Kringstad et al. 2019). Stang et al. demonstrated comparable proportions to our patient cohort, with the primary reasons for emergency department visits being lacerations, followed by hand fractures and infections (Plotsker et al. 2022).

In a report from the German Hand Trauma Register spanning from 2019 to 2021, a comprehensive analysis was conducted on a total of 12,988 patients from 57 clinics across the country. The reported mean age in this dataset was slightly greater than that in our patient cohort (44.3 years compared to 40.1 years). Additionally, our patient cohort exhibited a slightly higher percentage of females (36% compared to 28%). Furthermore, it is noteworthy that since the registry was established only in 2018, not all medical centers, particularly pediatric ones, are encompassed in the registry. As a result, differences in patient demographics and characteristics may exist between our dataset and the registry, as our study covered 16 years. The same disparities could be seen in an epidemiological analysis from Germany conducted by Stang et al. (Plotsker et al. 2022). Moreover, it is important to note that trends in different injuries could not be directly compared to our data. This is because, since the inception of the registry, the number of participating centers has increased from 44 to 57; therefore, the annual number of injuries increased from 1928 to 6580 (Nyszkiewicz et al. 2020).

The incidence of superficial lacerations, deep lacerations, metacarpal and finger fractures, wrist fractures, joint dislocations, sprains and strains as well as burns and corrosions increased significantly in our patient cohort over the study period. The most substantial increases were noted among working-age adults. The highest numbers of injuries in this patient group were also reported by other authors (Keller et al. 2012; Kringstad et al. 2019; Plotsker et al. 2022; Ambulantes 2022). Therefore, prioritizing workplace safety remains paramount as one of the most crucial preventive measures to further diminish hand trauma rates. Noteworthy reductions in amputation rates were observed, particularly in all age groups except for 50–59 years, 70–79 years, and 80–89 years. However, significance was observed only in the age groups of 0–9 years and 30–39 (Supplementary Table 2). A 10-year analysis in the USA revealed a slight decrease in the number of amputations, although the difference was not statistically significant (Kringstad et al. 2019). Furthermore, a 16-year analysis of patients in the United Kingdom unveiled a notable rise in the incidence of amputations, escalating from 2562 cases in 1998–1999 to 3495 cases in 2014–2015 (Keller et al. 2012). Our data align with the published global trends of hand amputation injuries, indicating that in high-income countries, the rates of such injuries tend to decrease (Ambulantes 2022). This trend can be attributed to the heightened emphasis on workplace safety, the improved socioeconomic status of the majority of the population, and increased attention to the safety of consumer products and the home environment, particularly concerning children (Hoefer 2022; Crowe et al. 2020).

The World Health Organization highlights substantial global progress in improving life expectancy and survival rates over the past decades. In Europe, the probability of a newborn surviving to 60 years of age in 1950 was nearly 70%, while worldwide it was 46%. By 2019, in the Americas and Europe, the probability of survival to 60 years had reached 87% and 89%, respectively (World Health Organization 2024). It is anticipated that the growth of the population aged 67 and older will continue, with the number projected to reach 20.9 million globally by 2048, mainly due to the high probability of survival to 60 years in the Americas and Europe (World Health Organization 2023, 2024). According to the Federal Statistical Office of Germany, individuals aged 65 and older represent an increasingly larger proportion of the total population over time. This proportion rose from 15% in 1991 to 22% in 2022 (Statistisches Bundesamt 2019). Moreover, it reports that the number of people of working age is expected to decrease by 7.2 million, while the number of those aged 67 and older is projected to increase by 2.4 million in 2035 (Bundesamt and (Destatis) 2021). Additionally, men in the year 2035 would have an average life expectancy at birth of 80.2 years, and women would have 84.3 years, corresponding to the prognosis in our region (Bundesamt and (Destatis) 2021). Life expectancy at birth in our region is projected to increase by 1.7 years for men to 80.2 years and by 1.3 years for women to 84.4 years by 2030. Particularly noteworthy is the 55.3% increase in the population aged 85 and above in the central region by 2030, with an even larger increase of 71.1% in the surrounding areas (Landeshauptstadt Hannover und Region Hannover 2020). In our analysis, we observed a steady rise in admissions, showing an average annual increase of approximately 2%. According to our analysis, individuals aged 60 and above accounted for approximately 20% of all patients presenting in the emergency department annually. As previously mentioned, hand injuries in elderly patients often exhibit greater severity and necessitate more complex treatment procedures (Plotsker et al. 2022). Our analysis serves as a notable illustration of the challenges currently encountered and anticipated shortly, by hand trauma centers such as ours due to the global aging population.

There are a few limitations to this study. Firstly, it was conducted retrospectively, which may introduce inherent biases. Additionally, our analysis relied on written and coded diagnoses, which are subject to the quality of medical documentation. While grouping injuries into broad categories can help mitigate coding errors, it also limits the level of detail available for analysis. Furthermore, the lack of access to medical records from the primary care sector prevented us from analyzing important factors such as comorbidities and medications that could influence injury trends. In our study, we did not analyze specific work-related injuries, as our focus was on the general trends of all patients who presented to our emergency department. However, this kind of analysis could allow us to determine possible changes in safety in work-related environments. We performed a single-center analysis, which may not represent the current trends in other hand trauma centers. However, our findings parallel those presented in the German Hand Trauma Register, which, regrettably, was established only in 2018. Despite this alignment, there are still some minor differences worth noting. Moreover, the collection of data from current hand trauma registries should consider the burden of increasing numbers of visits to emergency departments with minor hand and wrist injuries, which are not clearly represented in the current registry. Patients who disagreed to participate were included in the registry. Further studies should try to focus on possible associations between comorbidities and cofactors of specific injuries to better understand the mechanisms of injuries and initiate specific prevention programs. Moreover, more high-volume centers should be included to better understand the possible influence of smaller clinics and private medical centers on the care of patients with various types of hand trauma.

## Conclusions

In summary, our analysis revealed a notable annual increase in both the total number of cases and the average age of patients over the past two decades. Moreover, the number of presentations consistently and significantly increased annually by an average of 2%. Of particular concern is the increasing number of patients with minor injuries, including superficial lacerations, deep lacerations, metacarpal and finger fractures, sprains, strains, and joint dislocations. The majority of these patients could be effectively treated in smaller clinics or private medical centers. However, an identified demographic shift toward an aging population may result in a significant increase in patients with comorbidities. This could necessitate hospital admissions, placing greater demands on financial and personnel resources. Our study highlights the ongoing necessity of adapting the structure of the healthcare system and reimbursement policies for outpatient care for hand injuries. A particular focus should be on prioritizing the decentralized care of patients with minor hand injuries, and increasing attention should be given to preventive strategies, especially for elderly patients.

### Supplementary Information

Supplementary Material 1.

Supplementary Material 2.

## Data Availability

The datasets used and/or analyzed during the current study are available from the corresponding author upon reasonable request.
