# Supplementary material for: Demographic shifts reshaping the landscape of hand trauma: a comprehensive single-center analysis of changing trends in hand injuries from 2007 to 2022
Source: Inj Epidemiol. 2024 Jun 13;11:25. doi: 10.1186/s40621-024-00510-8 (PMC11170831; doi:10.1186/s40621-024-00510-8)
Supplement: Supplementary file 1 — Supplementary Material 1. [file 40621_2024_510_MOESM1_ESM.docx]

Supplementary Table 1. Trends of different types of injuries annually.

| **Injury type*** | **2007** | **2008** | **2009** | **2010** | **2011** | **2012** | **2013** | **2014** | **2015** | **2016** | **2017** | **2018** | **2019** | **2020** | **2021** | **2022** | **Total**** | **Change between EC and CC (%)***** | **P value** | **R²** | **P value****** |
| --- | --- | --- | --- | --- | --- | --- | --- | --- | --- | --- | --- | --- | --- | --- | --- | --- | --- | --- | --- | --- | --- |
| **Superficial lacerations** | 50  (4.55 %) | 50  (5.63 %) | 26  (3.11 %) | 51  (5.05 %) | 29  (2.70 %) | 52  (4.46 %) | 49  (5.95 %) | 62  (5.31 %) | 81  (5.78 %) | 69  (4.76 %) | 76  (5.12 %) | 124  (7.99 %) | 92  (6.03 %) | 99  (6.82 %) | 122  (8.61 %) | 139  (9.29 %) | 1171  (5.90 %) | +53.99 | **< 0.001** | 0.788 | **< 0.001** |
| **Deep lacerations** | 425  (38.71 %) | 354  (39.86 %) | 320  (38.28 %) | 375  (37.17 %) | 415  (38.57 %) | 448  (38.42 %) | 317  (38.47 %) | 413  (35.39 %) | 563  (40.19 %) | 618  (42.62 %) | 632  (42.62 %) | 628  (40.49 %) | 622  (40.76 %) | 638  (43.97 %) | 569  (40.16 %) | 611  (40.84 %) | 7948  (40.84 %) | +37.16 | **< 0.001** | 0.702 | **< 0.001** |
| **Complex hand injuries** | 165  (15.03 %) | 116  (13.06 %) | 86  (10.29 %) | 123  (12.19 %) | 144  (13.38 %) | 167  (14.32 %) | 100  (12.14 %) | 180  (15.42 %) | 198  (14.13 %) | 181  (12.48 %) | 165  (11.13 %) | 193  (12.44 %) | 166  (10.88 %) | 149  (10.27 %) | 142  (10.02 %) | 125  (8.36 %) | 2400  (12.10 %) | +18.04 | 0.068 | 0.09 | 0.258 |
| **Amputations** | 85  (7.74 %) | 77  (8.67 %) | 74  (8.85 %) | 81  (8.03 %) | 71  (6.60 %) | 89  (7.63 %) | 44  (5.34 %) | 76  (6.51 %) | 65  (4.64 %) | 59  (4.07 %) | 75  (5.06 %) | 56  (3.61 %) | 73  (4.78 %) | 63  (4.34 %) | 50  (3.53 %) | 48  (3.21 %) | 1086  (5.47 %) | -22.09 | **0.04** | 0.415 | **0.007** |
| **Wrist fractures** | 2  (0.18 %) | 7  (0.79 %) | 3  (0.36 %) | 6  (0.59 %) | 0  (0.00 %) | 5  (0.43 %) | 7  (0.85 %) | 4  (0.34 %) | 7  (0.50 %) | 7  (0.48 %) | 14  (0.94 %) | 6  (0.39 %) | 7  (0.46 %) | 10  (0.69 %) | 7  (0.49 %) | 9  (0.60 %) | 101  (0.51 %) | +49.25 | **0.003** | 0.364 | **0.013** |
| **Metacarpal and finger fractures** | 109  (9.93 %) | 87  (9.80 %) | 90  (10.77 %) | 106  (10.51 %) | 125  (11.62 %) | 116  (9.95 %) | 89  (10.80 %) | 127  (10.88 %) | 156  (11.13 %) | 132  (9.10 %) | 137  (9.24 %) | 183  (11.80 %) | 185  (12.12 %) | 163  (11.23 %) | 196  (13.83 %) | 244  (16.31 %) | 2245  (11.32 %) | +39.18 | **< 0.001** | 0.784 | **< 0.001** |
| **Joint dislocations** | 18  (1.64 %) | 10  (1.13 %) | 13  (1.56 %) | 28  (2.78 %) | 29  (2.70 %) | 24  (2.06 %) | 15  (1.82 %) | 35  (3.00 %) | 54  (3.85 %) | 55  (3.79 %) | 47  (3.17 %) | 31  (2.00 %) | 40  (2.62 %) | 46  (3.17 %) | 26  (1.83 %) | 54  (3.61 %) | 525  (2.65 %) | +51.28 | **< 0.001** | 0.473 | **0.003** |
| **Sprains and strains** | 8  (0.73 %) | 7  (0.79 %) | 7  (0.84 %) | 2  (0.20 %) | 3  (0.28 %) | 8  (0.69 %) | 6  (0.73 %) | 5  (0.43 %) | 9  (0.64 %) | 16  (1.10 %) | 12  (0.81 %) | 12  (0.77 %) | 17  (1.11 %) | 18  (1.24 %) | 43  (3.03 %) | 29  (1.94 %) | 202  (1.02 %) | +70.51 | **0.004** | 0.586 | **< 0.001** |
| **Phlegmon of hand** | 149  (13.57 %) | 95  (10.70 %) | 127  (15.19 %) | 130  (12.88 %) | 153  (14.22 %) | 156  (13.38 %) | 127  (15.41 %) | 175  (15.00 %) | 157  (11.21 %) | 184  (12.69 %) | 178  (12.00 %) | 180  (11.61 %) | 194  (12.71 %) | 151  (10.41 %) | 145  (10.23 %) | 113  (7.55 %) | 2414  (12.17 %) | +14.59 | 0.084 | 0.127 | 0.175 |
| **Hand and wrist tenosynovitis** | 8  (0.73 %) | 5  (0.56 %) | 6  (0.72 %) | 8  (0.79 %) | 8  (0.74 %) | 12  (1.03 %) | 11  (1.33 %) | 19  (1.63 %) | 13  (0.93 %) | 8  (0.55 %) | 12  (0.81 %) | 12  (0.77 %) | 6  (0.39 %) | 10  (0.69 %) | 9  (0.64 %) | 7  (0.47 %) | 154  (0.78 %) | 0 | 1.000 | 0.023 | 0.574 |
| **Acute joint inflammations** | 2  (0.18 %) | 1  (0.11 %) | 3  (0.36 %) | 2  (0.20 %) | 5  (0.46 %) | 4  (0.34 %) | 1  (0.12 %) | 0  (0.00 %) | 3  (0.21 %) | 3  (0.21 %) | 3  (0.20 %) | 1  (0.06 %) | 4  (0.26 %) | 2  (0.14 %) | 3  (0.21 %) | 4  (0.27 %) | 41  (0.21 %) | +21.74 | 0.189 | 0.04 | 0.458 |
| **Burns and corrosions** | 77  (7.01 %) | 79  (8.90 %) | 81  (9.69 %) | 97  (9.61 %) | 94  (8.74 %) | 85  (7.29 %) | 58  (7.04 %) | 71  (6.08 %) | 95  (6.78 %) | 118  (8.14 %) | 132  (8.90 %) | 125  (8.06 %) | 120  (7.86 %) | 102  (7.03 %) | 105  (7.41 %) | 113  (7.55 %) | 1552  (7.82 %) | +29.45 | **< 0.001** | 0.429 | **0.006** |

**Presented as number and percent of all injuries in one year; ** Presented as number and percent of all injuries in study period; ***change in frequency between the early cohort and the current cohort; ****P value for the significance of linear regression analysis;*

*.*
