# Supplementary material for: Demographic shifts reshaping the landscape of hand trauma: a comprehensive single-center analysis of changing trends in hand injuries from 2007 to 2022
Source: Inj Epidemiol. 2024 Jun 13;11:25. doi: 10.1186/s40621-024-00510-8 (PMC11170831; doi:10.1186/s40621-024-00510-8)
Supplement: Supplementary file 2 — Supplementary Material 2. [file 40621_2024_510_MOESM2_ESM.docx]

Supplementary Table 2. Annual distribution of amputations by age group.

|  | **2007** | **2008** | **2009** | **2010** | **2011** | **2012** | **2013** | **2014** | **2015** | **2016** | **2017** | **2018** | **2019** | **2020** | **2021** | **2022** | **Total** | **Change between EC and CC (%)*** | **P value** | **R²** | **P value**** |
| --- | --- | --- | --- | --- | --- | --- | --- | --- | --- | --- | --- | --- | --- | --- | --- | --- | --- | --- | --- | --- | --- |
| **0-9** | 6 | 4 | 8 | 6 | 6 | 13 | 7 | 4 | 5 | 3 | 7 | 1 | 3 | 6 | 4 | 4 | 87 | -38.89 | **0.048** | 0.161 | 0.124 |
| **10-19** | 11 | 10 | 4 | 5 | 12 | 8 | 1 | 4 | 3 | 2 | 6 | 1 | 2 | 9 | 3 | 4 | 85 | -45.45 | 0.083 | 0.241 | 0.054 |
| **20-29** | 14 | 12 | 6 | 12 | 6 | 5 | 3 | 9 | 6 | 11 | 16 | 8 | 7 | 5 | 5 | 3 | 128 | -8.96 | 0.717 | 0.156 | 0.130 |
| **30-39** | 13 | 8 | 19 | 15 | 5 | 9 | 5 | 11 | 4 | 6 | 7 | 5 | 7 | 5 | 8 | 3 | 130 | -47.06 | **0.016** | 0.389 | **0.010** |
| **40-49** | 8 | 16 | 13 | 13 | 16 | 20 | 12 | 16 | 11 | 5 | 10 | 15 | 14 | 6 | 15 | 5 | 195 | -28.95 | 0.057 | 0.111 | 0.208 |
| **50-59** | 18 | 15 | 8 | 14 | 10 | 15 | 9 | 18 | 20 | 8 | 21 | 10 | 20 | 15 | 9 | 14 | 224 | +9.35 | 0.600 | 0.001 | 0.901 |
| **60-69** | 7 | 7 | 12 | 11 | 10 | 13 | 5 | 10 | 10 | 12 | 6 | 8 | 10 | 11 | 2 | 8 | 142 | -10.67 | 0.515 | 0.054 | 0.385 |
| **70-79** | 7 | 4 | 4 | 3 | 5 | 3 | 2 | 4 | 3 | 11 | 2 | 3 | 6 | 6 | 3 | 5 | 71 | +21.88 | 0.462 | 0.002 | 0.883 |
| **80-89** | 1 | 1 | 0 | 2 | 1 | 3 | 0 | 0 | 3 | 1 | 0 | 4 | 4 | 0 | 1 | 2 | 23 | +87.50 | 0.227 | 0.050 | 0.406 |
| **90+** | 0 | 0 | 0 | 0 | 0 | 0 | 0 | 0 | 0 | 0 | 0 | 1 | 0 | 0 | 0 | 0 | 1 | -*** | -*** | -*** | -*** |
| **Total** | 85 | 77 | 74 | 81 | 71 | 89 | 44 | 76 | 65 | 59 | 75 | 56 | 73 | 63 | 50 | 48 | 1086 | -22.09 | **0.04** | 0.415 | **0.007** |

**change in frequency between the early cohort and the current cohort; **P value for the significance of linear regression analysis; ***not enough data for statistical analysis.*

Supplementary Table 3. Annual distribution of burns and corrosions by age group.

|  | **2007** | **2008** | **2009** | **2010** | **2011** | **2012** | **2013** | **2014** | **2015** | **2016** | **2017** | **2018** | **2019** | **2020** | **2021** | **2022** | **Total** | **Change between EC and CC (%)*** | **P value** | **R²** | **P value**** |
| --- | --- | --- | --- | --- | --- | --- | --- | --- | --- | --- | --- | --- | --- | --- | --- | --- | --- | --- | --- | --- | --- |
| **0-9** | 7 | 4 | 2 | 9 | 6 | 4 | 4 | 4 | 4 | 4 | 4 | 7 | 6 | 5 | 2 | 3 | 75 | -12.50 | 0.527 | 0.073 | 0.310 |
| **10-19** | 8 | 17 | 9 | 3 | 14 | 12 | 7 | 5 | 7 | 11 | 15 | 6 | 14 | 11 | 9 | 6 | 154 | +5.33 | 0.812 | 0.005 | 0.797 |
| **20-29** | 16 | 10 | 16 | 24 | 19 | 19 | 17 | 17 | 23 | 31 | 36 | 29 | 24 | 18 | 23 | 22 | 344 | +49.28 | **0.004** | 0.273 | **0.038** |
| **30-39** | 15 | 13 | 23 | 13 | 5 | 13 | 11 | 17 | 25 | 25 | 24 | 25 | 27 | 19 | 19 | 24 | 298 | +70.91 | **0.000** | 0.344 | **0.017** |
| **40-49** | 14 | 14 | 10 | 20 | 20 | 11 | 9 | 11 | 19 | 17 | 18 | 25 | 24 | 21 | 17 | 21 | 271 | +48.62 | **0.003** | 0.322 | **0.022** |
| **50-59** | 10 | 8 | 13 | 11 | 13 | 11 | 7 | 9 | 6 | 17 | 17 | 14 | 12 | 17 | 15 | 26 | 206 | +51.22 | **0.028** | 0.403 | **0.008** |
| **60-69** | 5 | 6 | 4 | 5 | 6 | 8 | 2 | 2 | 4 | 6 | 10 | 6 | 7 | 8 | 10 | 6 | 95 | +50.00 | **0.038** | 0.208 | 0.076 |
| **70-79** | 2 | 6 | 3 | 8 | 5 | 3 | 1 | 6 | 5 | 6 | 6 | 8 | 5 | 3 | 8 | 3 | 78 | +29.41 | 0.267 | 0.042 | 0.447 |
| **80-89** | 0 | 1 | 1 | 4 | 5 | 4 | 0 | 0 | 2 | 1 | 2 | 3 | 1 | 0 | 2 | 2 | 28 | -13.33 | 0.762 | 0.004 | 0.818 |
| **90+** | 0 | 0 | 0 | 0 | 1 | 0 | 0 | 0 | 0 | 0 | 0 | 2 | 0 | 0 | 0 | 0 | 3 | -*** | -*** | -*** | -*** |
| **Total** | 77 | 79 | 81 | 97 | 94 | 85 | 58 | 71 | 95 | 118 | 132 | 125 | 120 | 102 | 105 | 113 | 1552 | +29.45 | **< 0.001** | 0.429 | **0.006** |

**change in frequency between the early cohort and the current cohort; **P value for the significance of linear regression analysis; ***not enough data for statistical analysis.*

Supplementary Table 3. Annual distribution of deep lacerations by age group.

|  | **2007** | **2008** | **2009** | **2010** | **2011** | **2012** | **2013** | **2014** | **2015** | **2016** | **2017** | **2018** | **2019** | **2020** | **2021** | **2022** | **Total** | **Change between EC and CC (%)*** | **P value** | **R²** | **P value**** |
| --- | --- | --- | --- | --- | --- | --- | --- | --- | --- | --- | --- | --- | --- | --- | --- | --- | --- | --- | --- | --- | --- |
| **0-9** | 23 | 24 | 35 | 21 | 27 | 30 | 15 | 25 | 17 | 36 | 31 | 17 | 27 | 26 | 23 | 19 | 396 | -2.00 | 0.879 | 0.021 | 0.594 |
| **10-19** | 51 | 38 | 24 | 40 | 44 | 47 | 24 | 27 | 46 | 52 | 60 | 51 | 60 | 60 | 50 | 61 | 735 | +49.15 | **0.001** | 0.378 | **0.011** |
| **20-29** | 83 | 63 | 80 | 63 | 80 | 85 | 58 | 85 | 105 | 146 | 136 | 147 | 112 | 142 | 119 | 120 | 1624 | +72.03 | **0.000** | 0.598 | **<0.001** |
| **30-39** | 86 | 56 | 48 | 63 | 67 | 71 | 53 | 67 | 119 | 117 | 102 | 119 | 108 | 127 | 104 | 114 | 1421 | +78.08 | **0.000** | 0.610 | **<0.001** |
| **40-49** | 72 | 75 | 53 | 60 | 70 | 82 | 58 | 73 | 91 | 78 | 94 | 100 | 93 | 87 | 95 | 81 | 1262 | +32.41 | **0.000** | 0.490 | **0.003** |
| **50-59** | 56 | 52 | 39 | 62 | 58 | 68 | 45 | 68 | 87 | 92 | 106 | 90 | 102 | 95 | 89 | 106 | 1215 | +71.21 | **0.000** | 0.755 | **<0.001** |
| **60-69** | 31 | 25 | 23 | 40 | 33 | 33 | 37 | 43 | 52 | 52 | 57 | 49 | 67 | 54 | 55 | 55 | 706 | +66.42 | **0.000** | 0.781 | **<0.001** |
| **70-79** | 17 | 14 | 12 | 13 | 21 | 19 | 19 | 18 | 28 | 31 | 28 | 31 | 33 | 29 | 18 | 28 | 359 | +69.92 | **0.000** | 0.538 | **0.001** |
| **80-89** | 5 | 6 | 6 | 13 | 15 | 10 | 7 | 7 | 16 | 11 | 16 | 21 | 19 | 14 | 12 | 21 | 199 | +88.41 | **0.001** | 0.523 | **0.002** |
| **90+** | 1 | 1 | 0 | 0 | 0 | 3 | 1 | 0 | 2 | 3 | 2 | 3 | 1 | 4 | 4 | 6 | 31 | +316.67 | **0.003** | 0.571 | **<0.001** |
| **Total** | 425 | 354 | 320 | 375 | 415 | 448 | 317 | 413 | 563 | 618 | 632 | 628 | 622 | 638 | 569 | 611 | 7948 | +37.16 | **< 0.001** | 0.702 | **< 0.001** |

**change in frequency between the early cohort and the current cohort; **P value for the significance of linear regression analysis; ***not enough data for statistical analysis.*

Supplementary Table 4. Annual distribution of metacarpal and finger fractures by age group.

|  | **2007** | **2008** | **2009** | **2010** | **2011** | **2012** | **2013** | **2014** | **2015** | **2016** | **2017** | **2018** | **2019** | **2020** | **2021** | **2022** | **Total** | **Change between EC and CC (%)*** | **P value** | **R²** | **P value**** |
| --- | --- | --- | --- | --- | --- | --- | --- | --- | --- | --- | --- | --- | --- | --- | --- | --- | --- | --- | --- | --- | --- |
| **0-9** | 4 | 4 | 7 | 12 | 12 | 10 | 2 | 7 | 7 | 5 | 8 | 6 | 7 | 10 | 10 | 12 | 123 | +12.07 | 0.589 | 0.105 | 0.221 |
| **10-19** | 11 | 12 | 11 | 11 | 11 | 7 | 7 | 8 | 13 | 10 | 20 | 20 | 25 | 22 | 39 | 46 | 273 | +150.00 | **0.005** | 0.604 | **<0.001** |
| **20-29** | 20 | 8 | 13 | 14 | 21 | 17 | 19 | 27 | 27 | 31 | 25 | 32 | 32 | 26 | 35 | 34 | 381 | +74.10 | **0.000** | 0.766 | **<0.001** |
| **30-39** | 21 | 12 | 14 | 17 | 21 | 19 | 12 | 21 | 31 | 19 | 21 | 38 | 36 | 21 | 31 | 40 | 374 | +72.99 | **0.002** | 0.533 | **0.001** |
| **40-49** | 18 | 25 | 23 | 20 | 18 | 23 | 18 | 16 | 23 | 15 | 15 | 32 | 22 | 29 | 18 | 28 | 343 | +13.04 | 0.322 | 0.074 | 0.308 |
| **50-59** | 20 | 10 | 9 | 13 | 20 | 19 | 13 | 22 | 27 | 14 | 28 | 24 | 23 | 25 | 23 | 42 | 332 | +63.49 | **0.009** | 0.523 | **0.002** |
| **60-69** | 12 | 10 | 6 | 13 | 11 | 7 | 9 | 22 | 14 | 23 | 11 | 8 | 20 | 17 | 23 | 20 | 226 | +51.11 | **0.046** | 0.362 | **0.014** |
| **70-79** | 2 | 5 | 5 | 4 | 8 | 8 | 9 | 3 | 8 | 13 | 7 | 14 | 13 | 9 | 5 | 13 | 126 | +86.36 | **0.007** | 0.400 | **0.009** |
| **80-89** | 1 | 1 | 2 | 2 | 3 | 5 | 0 | 1 | 6 | 2 | 2 | 9 | 7 | 3 | 11 | 7 | 62 | +213.33 | **0.008** | 0.460 | **0.004** |
| **90+** | 0 | 0 | 0 | 0 | 0 | 1 | 0 | 0 | 0 | 0 | 0 | 0 | 0 | 1 | 1 | 2 | 5 | -*** | -*** | -*** | -*** |
| **Total** | 109 | 87 | 90 | 106 | 125 | 116 | 89 | 127 | 156 | 132 | 137 | 183 | 185 | 163 | 196 | 244 | 2245 | +39.18 | **< 0.001** | 0.784 | **< 0.001** |

**change in frequency between the early cohort and the current cohort; **P value for the significance of linear regression analysis; ***not enough data for statistical analysis.*

Supplementary Table 5. Annual distribution of sprains and strains by age group.

|  | **2007** | **2008** | **2009** | **2010** | **2011** | **2012** | **2013** | **2014** | **2015** | **2016** | **2017** | **2018** | **2019** | **2020** | **2021** | **2022** | **Total** | **Change between EC and CC (%)*** | **P value** | **R²** | **P value**** |
| --- | --- | --- | --- | --- | --- | --- | --- | --- | --- | --- | --- | --- | --- | --- | --- | --- | --- | --- | --- | --- | --- |
| **0-9** | 0 | 0 | 1 | 0 | 1 | 0 | 0 | 0 | 1 | 0 | 0 | 0 | 0 | 1 | 2 | 1 | 7 | -*** | -*** | -*** | -*** |
| **10-19** | 0 | 2 | 0 | 0 | 1 | 0 | 1 | 2 | 2 | 0 | 1 | 6 | 6 | 4 | 10 | 5 | 40 | +466.67 | **0.011** | 0.566 | **<0.001** |
| **20-29** | 5 | 2 | 1 | 1 | 1 | 4 | 1 | 0 | 3 | 8 | 4 | 3 | 7 | 4 | 11 | 7 | 62 | +213.33 | **0.004** | 0.384 | **0.010** |
| **30-39** | 3 | 1 | 0 | 0 | 0 | 1 | 2 | 1 | 0 | 3 | 1 | 0 | 0 | 3 | 11 | 6 | 32 | +200.00 | 0.179 | 0.253 | **0.047** |
| **40-49** | 0 | 1 | 3 | 0 | 0 | 3 | 2 | 0 | 1 | 0 | 2 | 0 | 2 | 1 | 3 | 4 | 22 | +44.44 | 0.481 | 0.137 | 0.158 |
| **50-59** | 0 | 0 | 2 | 0 | 0 | 0 | 0 | 1 | 0 | 3 | 1 | 2 | 1 | 3 | 3 | 3 | 19 | +433.33 | **0.006** | 0.516 | **0.002** |
| **60-69** | 0 | 1 | 0 | 1 | 0 | 0 | 0 | 1 | 1 | 2 | 0 | 1 | 0 | 1 | 2 | 3 | 13 | +233.33 | 0.051 | 0.315 | **0.024** |
| **70-79** | 0 | 0 | 0 | 0 | 0 | 0 | 0 | 0 | 0 | 0 | 2 | 0 | 1 | 1 | 1 | 0 | 5 | -*** | -*** | -*** | -*** |
| **80-89** | 0 | 0 | 0 | 0 | 0 | 0 | 0 | 0 | 1 | 0 | 1 | 0 | 0 | 0 | 0 | 0 | 2 | -*** | -*** | -*** | -*** |
| **90+** | 0 | 0 | 0 | 0 | 0 | 0 | 0 | 0 | 0 | 0 | 0 | 0 | 0 | 0 | 0 | 0 | 0 | -*** | -*** | -*** | -*** |
| **Total** | 8 | 7 | 7 | 2 | 3 | 8 | 6 | 5 | 9 | 16 | 12 | 12 | 17 | 18 | 43 | 29 | 202 | +70.51 | **0.004** | 0.586 | **< 0.001** |

**change in frequency between the early cohort and the current cohort; **P value for the significance of linear regression analysis; ***not enough data for statistical analysis.*

Supplementary Table 6. Annual distribution of superficial lacerations by age group.

|  | **2007** | **2008** | **2009** | **2010** | **2011** | **2012** | **2013** | **2014** | **2015** | **2016** | **2017** | **2018** | **2019** | **2020** | **2021** | **2022** | **Total** | **Change between EC and CC (%)*** | **P value** | **R²** | **P value**** |
| --- | --- | --- | --- | --- | --- | --- | --- | --- | --- | --- | --- | --- | --- | --- | --- | --- | --- | --- | --- | --- | --- |
| **0-9** | 0 | 7 | 6 | 3 | 3 | 9 | 2 | 1 | 4 | 6 | 4 | 8 | 8 | 6 | 4 | 5 | 76 | +45.16 | 0.186 | 0.082 | 0.283 |
| **10-19** | 0 | 6 | 5 | 8 | 4 | 12 | 4 | 6 | 6 | 9 | 18 | 13 | 10 | 19 | 24 | 21 | 165 | +166.67 | **0.003** | 0.699 | **<0.001** |
| **20-29** | 0 | 4 | 4 | 5 | 7 | 7 | 6 | 8 | 16 | 14 | 11 | 27 | 20 | 14 | 32 | 34 | 209 | +309.76 | **0.000** | 0.788 | **<0.001** |
| **30-39** | 0 | 7 | 4 | 7 | 2 | 8 | 9 | 17 | 16 | 11 | 10 | 22 | 20 | 13 | 21 | 25 | 192 | +155.56 | **0.002** | 0.750 | **<0.001** |
| **40-49** | 0 | 8 | 2 | 10 | 3 | 6 | 8 | 12 | 16 | 9 | 15 | 22 | 10 | 16 | 16 | 16 | 169 | +144.90 | **0.001** | 0.624 | **<0.001** |
| **50-59** | 0 | 8 | 1 | 10 | 0 | 3 | 9 | 7 | 10 | 12 | 12 | 15 | 15 | 15 | 12 | 24 | 153 | +202.63 | **0.000** | 0.704 | **<0.001** |
| **60-69** | 5 | 6 | 4 | 5 | 5 | 3 | 8 | 6 | 5 | 6 | 3 | 7 | 6 | 9 | 11 | 7 | 96 | +28.57 | 0.159 | 0.307 | **0.026** |
| **70-79** | 2 | 1 | 0 | 3 | 3 | 2 | 3 | 4 | 2 | 2 | 1 | 7 | 2 | 2 | 0 | 4 | 38 | +11.11 | 0.781 | 0.039 | 0.465 |
| **80-89** | 5 | 2 | 0 | 0 | 2 | 2 | 0 | 1 | 5 | 0 | 2 | 3 | 1 | 2 | 1 | 2 | 28 | +33.33 | 0.543 | 0.005 | 0.793 |
| **90+** | 0 | 1 | 0 | 0 | 0 | 0 | 0 | 0 | 1 | 0 | 0 | 0 | 0 | 3 | 1 | 1 | 7 | -*** | -*** | -*** | -*** |
| **Total** | 12 | 50 | 26 | 51 | 29 | 52 | 49 | 62 | 81 | 69 | 76 | 124 | 92 | 99 | 122 | 139 | 1133 | +53.99 | **< 0.001** | 0.788 | **< 0.001** |

**change in frequency between the early cohort and the current cohort; **P value for the significance of linear regression analysis; ***not enough data for statistical analysis.*

Supplementary Table 7. Annual distribution of wrist fractures by age group.

|  | **2007** | **2008** | **2009** | **2010** | **2011** | **2012** | **2013** | **2014** | **2015** | **2016** | **2017** | **2018** | **2019** | **2020** | **2021** | **2022** | **Total** | **Change between EC and CC (%)*** | **P value** | **R²** | **P value**** |
| --- | --- | --- | --- | --- | --- | --- | --- | --- | --- | --- | --- | --- | --- | --- | --- | --- | --- | --- | --- | --- | --- |
| **0-9** | 0 | 0 | 0 | 0 | 0 | 0 | 0 | 0 | 0 | 0 | 0 | 0 | 0 | 0 | 0 | 0 | 0 | -*** | -*** | -*** | -*** |
| **10-19** | 0 | 0 | 0 | 1 | 0 | 1 | 2 | 0 | 0 | 1 | 1 | 0 | 2 | 0 | 3 | 2 | 13 | +125.00 | 0.213 | 0.286 | **0.033** |
| **20-29** | 1 | 0 | 2 | 2 | 0 | 0 | 3 | 1 | 6 | 3 | 2 | 3 | 2 | 2 | 1 | 0 | 28 | +111.11 | 0.114 | 0.023 | 0.575 |
| **30-39** | 1 | 4 | 1 | 0 | 0 | 0 | 1 | 2 | 1 | 2 | 3 | 0 | 0 | 2 | 1 | 5 | 23 | +55.56 | 0.425 | 0.052 | 0.396 |
| **40-49** | 0 | 1 | 0 | 1 | 0 | 0 | 1 | 1 | 0 | 1 | 1 | 1 | 2 | 1 | 1 | 0 | 11 | +75.00 | 0.224 | 0.130 | 0.170 |
| **50-59** | 0 | 0 | 0 | 1 | 0 | 1 | 0 | 0 | 0 | 0 | 3 | 1 | 0 | 3 | 1 | 1 | 11 | +350.00 | 0.084 | 0.227 | 0.062 |
| **60-69** | 0 | 2 | 0 | 0 | 0 | 2 | 0 | 0 | 0 | 0 | 3 | 1 | 1 | 1 | 0 | 1 | 11 | +75.00 | 0.447 | 0.024 | 0.566 |
| **70-79** | 0 | 0 | 0 | 1 | 0 | 1 | 0 | 0 | 0 | 0 | 1 | 0 | 0 | 0 | 0 | 0 | 3 | -*** | -*** | -*** | -*** |
| **80-89** | 0 | 0 | 0 | 0 | 0 | 0 | 0 | 0 | 0 | 0 | 0 | 0 | 0 | 1 | 0 | 0 | 1 | -*** | -*** | -*** | -*** |
| **90+** | 0 | 0 | 0 | 0 | 0 | 0 | 0 | 0 | 0 | 0 | 0 | 0 | 0 | 0 | 0 | 0 | 0 | -*** | -*** | -*** | -*** |
| **Total** | 2 | 7 | 3 | 6 | 0 | 5 | 7 | 4 | 7 | 7 | 14 | 6 | 7 | 10 | 7 | 9 | 101 | +49.25 | **0.003** | 0.364 | **0.013** |

**change in frequency between the early cohort and the current cohort; **P value for the significance of linear regression analysis; ***not enough data for statistical analysis.*
